# The impact of orthotopic neobladder vs ileal conduit urinary diversion after cystectomy on the survival outcomes in patients with bladder cancer: A propensity score matched analysis

**DOI:** 10.1002/cam4.3404

**Published:** 2020-09-01

**Authors:** Xiaohong Su, Kaihui Wu, Shuo Wang, Wei Su, Chuanyin Li, Bingkun Li, Xiangming Mao

**Affiliations:** ^1^ Department of Urology Zhujiang Hospital Southern Medical University Guangzhou China

**Keywords:** bladder cancer, ileal conduit, orthotopic neobladder, radical cystectomy, SEER

## Abstract

**Background:**

Bladder cancer (BCa) is the most common urinary malignancy. The standard surgical treatment for patients with muscle‐invasive BCa is cystectomy plus urinary diversion. Ileal conduit (IC) or orthotopic neobladder (ON), which have different indications, are the most commonly performed urinary diversions.

**Methods and materials:**

We sampled 5480 BCa patients from the Surveillance, Epidemiology, and End Results (SEER) database from 2004 to 2015. Kaplan‐Meier method with the log‐rank test was used to assess cancer‐specific survival (CSS) and overall survival (OS). Univariate and multivariate Cox's proportional hazard model was conducted to evaluate the hazard ratio of cancer‐specific mortality and all‐cause mortality before and after propensity score matching (PSM).

**Results:**

We identified 5480 patients who received radical cystectomy (RC) plus IC (n = 5071) or ON (n = 409) with a median follow‐up period of 33 months (interquartile range, 13‐78 months). Patients in the ON group tended to be male and younger, with a higher percentage of married individuals, early pathological T stage, lymphadenectomy, and non‐radiotherapy (all *P* < .05). After 1:1 PSM, 409 matched pairs were selected. Univariate and multivariate analysis showed that the ON group had better CSS and OS probabilities than the IC group in the overall cohort [hazard ratio (HR): 0.692, 95% confidence intervals (CI): 0.576‐0.831, *P* < .001; HR: 0.677, 95% CI: 0.579‐0.793, *P* < .001 respectively]. However, subgroup analysis revealed that only patients with pathological T2 stage benefited from ON diversion after PSM in the context of CSS (*P* = .016) and OS (*P* <.001).

**Conclusions:**

Young, married, and male patients with early pathological T stage, especially T2 stage, were more suitable to receive RC plus ON surgery, which could improve their probability of survival.

## Graphical abstract

Young, married, and male patients with early pathological T stage, especially T2 stage, were more suitable to receive RC plus ON surgery, which might improve the survival probabilities.

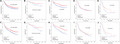

## 1 INTRODUCTION

Bladder cancer (BCa) is the most common malignancy in the urinary system and the 4th most commonly diagnosed cancer in males in the United States.^1^ According to the latest European Association of Urology guideline on muscle‐invasive bladder cancer (MIBC), radical cystectomy (RC) with urinary diversion is the standard treatment recommended for patients with MIBC T2‐T4a, N0‐Nx, M0, and selected patients with high‐risk nonmuscle‐invasive bladder cancer (NMIBC).^2^ Urinary diversion is the second important step after RC. There are currently three alternative types of urinary diversion, including abdominal diversion, urethral diversion, and rectosigmoid diversion. Age, comorbidity, cardio‐pulmonary function, cognitive function, patient's social support, and preference are all important factors that should be considered when choosing the type of urinary diversion. Nowadays, the most commonly performed urinary diversions are ileal conduit (IC) or orthotopic neobladder (ON).^2^, ^3^ Previous studies have aimed to assess the impact of IC and ON diversions on the health‐related quality‐of‐life (HRQoL) of BCa patients. Although no significant differences in QoL were observed between continent and incontinent urinary diversion, ON was shown to provide a better body image and normal urethral voiding.

In a multicenter study that included 2501 patients undergoing RC for BCa, regardless of the urinary diversion that was used, the actuarial 5‐year cancer‐specific survival (CSS) and overall survival (OS) were 67% and 47% respectively.^4^ Oncological outcomes after RC depend on specific variables, including tumor stage, lymph node yield and positivity, lymphovascular invasion (LVI), surgical margin status, and neoadjuvant chemotherapy. Nevertheless, Yossepowitch and his colleagues reported that ON diversion did not compromise the oncological outcomes of cystectomy, because no CSS or recurrence‐free survival (RFS) differences were identified between the ON (n = 214) and IC (n = 269) cohorts when stratified by pathological stage.^5^ To the best of our knowledge, there have been no randomized controlled trials or retrospective studies on a large population aimed at comparing the oncological outcomes between IC and ON diversion. Consequently, the aim of this study is to use data from the Surveillance, Epidemiology, and End Results (SEER) database, which represents 28% of the United States’ population, to compare the CSS and OS of patients with IC or ON diversion. Ultimately, the aim is to provide a relatively good level of evidence for urologists and patients to make the decision about which urinary diversion to use.

## 2 MATERIALS AND METHODS

### 2.1 Data source and study population

**FIGURE 1:**
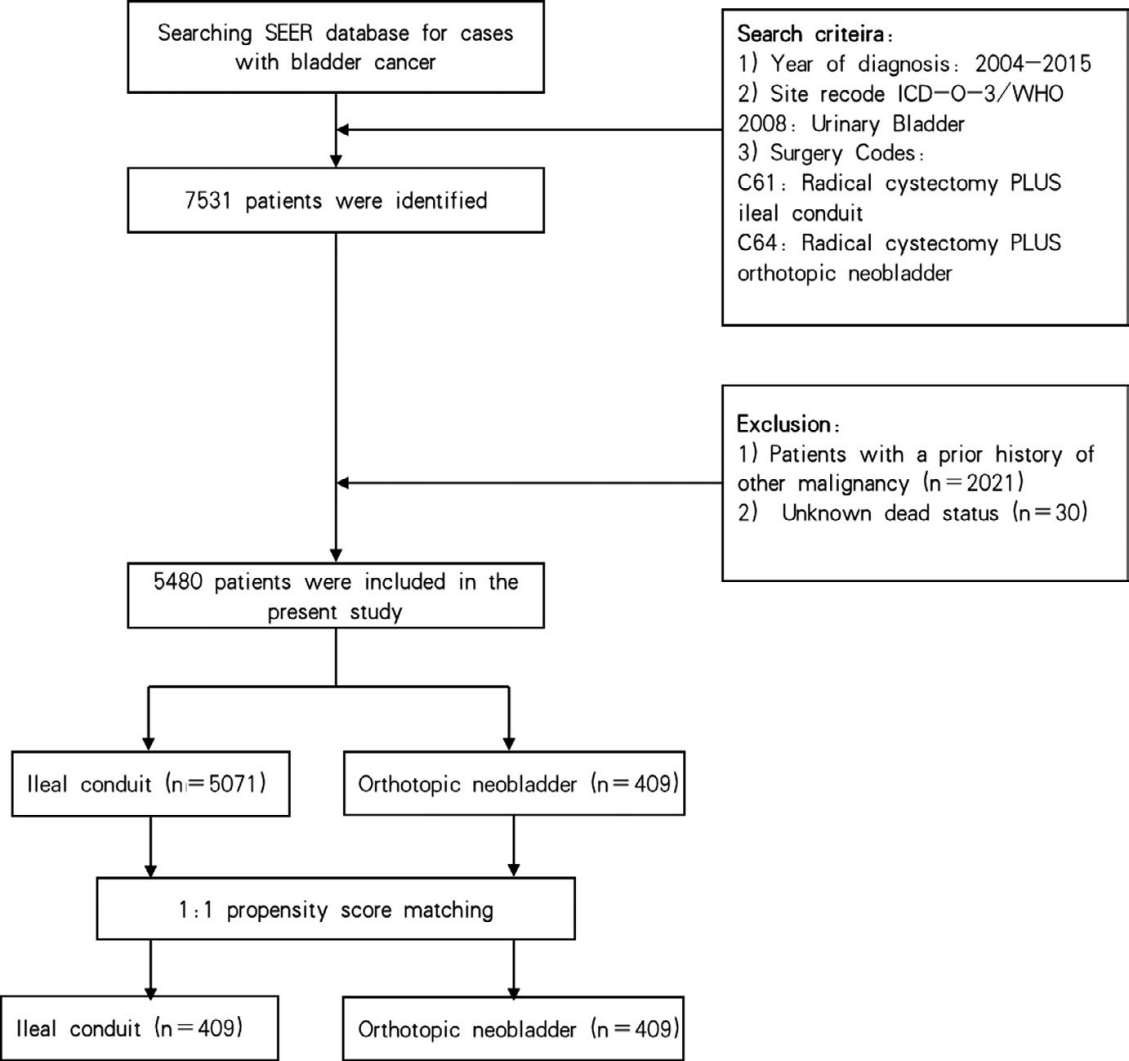
Flow‐chart demonstrating the approach used to identify patients with bladder cancer registered in the SEER database from 2004 to 2015

The study cases in this study were retrieved from 18 SEER cancer registries using SEER*STAT 8.3.6 software. Patients were included if they met the following criteria (n = 7531): (a) year of diagnosis between 2004 and 2015; (b) using the International Classification of Diseases for Oncology 3/WHO site recode: C67.0‐67.9 (urinary bladder); (c) using the surgery codes C61 and C64 (radical cystectomy plus IC or ON). The following exclusion criteria were also applied: (a) patients with a prior history of other malignancy (n = 2021); (b) unknown death status (n = 30) (Figure 1). A total of 5480 patients were included and their clinicopathological data were retrospectively analyzed.

### 2.2 Variables definition

Demographic characteristics included age at diagnosis, gender, marital status, and race. Tumor characteristics included tumor location, histology, TNM status, stage, grade, LVI, and lymphadenectomy. Treatment information included surgery approach, radiotherapy, and chemotherapy. Marital status was defined as married, unmarried, or unknown. Unmarried included never married, separated/divorced, and widowed patients. Race was categorized as white, black, Asian/Pacific Islander, and American Indian/Alaskan Native. Tumor location was defined as primary tumor located in the trigone, dome, lateral wall, anterior wall, posterior wall, bladder neck, ureteric orifice, urachus, overlapping lesions, and other locations not otherwise specified of the bladder. BCa histology was classified as transitional cell carcinoma, squamous cell carcinoma, and other rare types. Pathological TNM status was assessed according to the American Joint Committee on Cancer, sixth edition. T stages were divided into subgroups as T0‐Tis‐Ta‐T1, T2, T3, T4, and Tx/NA for further analysis. Tumor grade was assigned according to the SEER database as grades I, II, III, IV, and unknown. According to the “RX Summ‐Scope Reg LN Sur (2003+)” column in the SEER database, lymphadenectomy included 1 to 3 removed regional lymph nodes, 4 or more removed regional lymph nodes, and unknown number of removed regional lymph nodes. Surgical approach was categorized as RC plus ID or ON according to the “RX Summ‐Surg Prim Site (1998+).”

### 2.3 Statistical analyses

SPSS version 23.0 (IBM Corp, Armonk, NY) was used for all statistical analyses and propensity score matching (PSM). Chi‐square test and Mann‐Whitney *U* tests were used to determine the significant differences between categorical and continuous variables and surgical approach. The CSS and OS curves were created by the Kaplan‐Meier method with the log‐rank test. To estimate the impact of IC or ON on prognosis, univariate and multivariate cox regression analysis were carried out, and the results were presented as hazards ratios (HR) and 95% confidence intervals (95% CI). A fewer number of patients were treated by ON than by IC diversion (409 vs 5071). Moreover, there were also imbalances in baseline characteristics, such as age, gender, marital status, T stage, lymphadenectomy and radiotherapy; therefore, PSM was used for more objective comparison. For PSM, patients receiving IC or ON were matched 1:1 with a caliper set at 0. The matching algorithm was nearest neighbor matching, and the estimation algorithm was logistic regression. The PSM was described and adjusted for different variables, including age, gender, sex, marital status, TNM stages, lymphadenectomy, radiotherapy, and chemotherapy. All reported *P*‐values were two‐tailed and a *P* < .050 was considered significant.

## 3 RESULTS

### 3.1 Comparison of baseline clinicopathological characteristics before and after PSM

A total of 5480 BCa patients who underwent RC plus IC (n = 5071) or ON (n = 409) were included (Table S1). There were no significant differences in terms of race, primary tumor site, histology, N stage, M stage, grade, and chemotherapy between the two different urinary diversions (all *P* > .050). However, patients in the ON group tended to be younger (median year: 63 vs 69, *P* < .001) and male (97.1% vs 86.9%, *P* < .001), with a higher percentage of married individuals (73.3% vs 64.1%, *P* < .001), early T stage (T0‐T2: 59.6% vs 49.3%, *P* < .001), lymphadenectomy (97.1% vs 91.3%, *P* < .001), and nonradiotherapy (98.5% vs 96.1%, *P* = .012) before PSM. After 1:1 PSM adjusting for age, gender, sex, marital status, TNM stages, lymphadenectomy, radiotherapy, and chemotherapy, there were no significant differences between the IC and ON groups (all *P* > .050, Table 1).

**TABLE 1:** Clinical and pathological characteristics of BCa patients between different urinary diversion after PSM

Characteristic	Urinary diversion, % of patients		Chi‐square or Z	*P* value
	Ileal conduit (n = 409)	Orthotopic neobladder (n = 409)		
Age, median (IQR)	63 (56‐69)	63 (55‐69)	−0.789	.430
Gender			0.440	.507
Male	97.8	97.1		
Female	2.2	2.9		
Race			7.585	.108
White	90.2	89.7		
Black	5.6	2.9		
AI	0.5	0.5		
API	3.4	6.6		
Unknown	0.2	0.2		
Marital status			3.743	.154
Married	73.1	73.3		
No	26.7	25.2		
Unknown	0.2	1.5		
Primary tumor site			11.331	.254
Trigone of bladder	6.8	3.9		
Dome of bladder	5.4	3.7		
Lateral wall of bladder	17.1	17.1		
Anterior wall of bladder	1.2	2.4		
Posterior wall of bladder	7.3	7.8		
Bladder neck	1.7	3.7		
Ureteric orifice	1.2	1.5		
Urachus	0.0	0.5		
Overlapping lesion of bladder	20.0	20.0		
Bladder, NOS	39.1	39.4		
Histology			1.580	.454
Transitional cell carcinoma	94.4	92.2		
Squamous cell carcinoma	2.0	2.7		
Other types	3.7	5.1		
Pathological T stage			0.168	.997
T0‐Tis‐Ta‐T1	15.2	14.4		
T2	44.7	45.2		
T3	27.6	28.4		
T4	11.5	11.0		
Tx/NA	1.0	1.0		
Pathological N stage			3.525	.317
N0	81.2	79.0		
N1	11.0	9.5		
N2	6.6	10.0		
N3	0.0	0.0		
Nx/NA	1.2	1.5		
Pathological M stage			4.328	.115
M0	96.6	94.6		
M1	2.0	4.4		
Mx/NA	1.5	1.0		
Grade			4.916	.296
I	0.7	0.7		
II	4.6	4.2		
III	26.9	34.0		
IV	63.1	57.2		
Unknown	4.6	3.9		
Lymphadenectomy			2.687	.261
No	2.7	2.7		
Yes	96.1	97.1		
Others	1.2	0.2		
Adjuvant radiation			2.020	.155
No	99.5	98.5		
Yes	0.5	1.5		
Adjuvant chemotherapy			0.127	.721
No	60.6	59.4		
Yes	39.4	40.6		

Abbreviations: AI, American Indian/Alaskan Native; API, Asian/Pacific Islander; BCa, bladder cancer; IQR, interquartile range; NA, not available; NOS, not otherwise specified; PSM, propensity score matching.

### 3.2 Univariate and multivariate analyses of the predictors of cancer‐specific mortality and all other‐cause mortality before PSM

**FIGURE 2:**
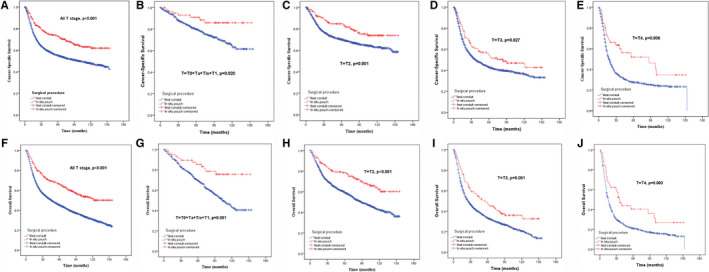
Influence of the urinary diversion on Cancer‐Specific Survival (A‐E) and Overall Survival (F‐J) in Bladder Cancer patients stratified by pathological T stage before propensity score matching

The median follow‐up period was 33 months (interquartile range: 33‐78 months), with a total of 3034 (59.8%) and 170 (41.6%) deaths in the IC and ON groups respectively. Among all of the deaths, 2178 (43%) and 127 (31.1%) patients died specifically from BCa in the IC and ON groups respectively. The 5‐year CSS and OS probabilities for the IC group and the ON group were 55%, 72.9%, and 45.1%, 66.0% respectively (Figure 2A and F, both *P* < .001). When stratified by T stages, patients with each T stage in the ON group also showed longer CSS and OS than those in the IC group (Figure 2B‐2E and G‐J).

Before PSM, univariate analyses revealed that age, marital status, primary tumor site, histology, TNM stages, grade, lymphadenectomy, urinary diversion, and radiotherapy were all associated with cancer‐specific mortality (CSM) and all‐cause mortality (ACM) (Tables S2 and S3). After controlling for other factors, urinary diversion was an independent risk factor for CSM and ACM in the multivariate analyses [ON vs IC, HR: 0.692, 95% CI: 0.576‐0.831, *P* < .001; HR: 0.677, 95% CI: 0.579‐0.793, *P* < .001 respectively] (Tables S2 and S3).

### 3.3 Impact of IC vs ON on CSS and OS after PSM

**FIGURE 3:**
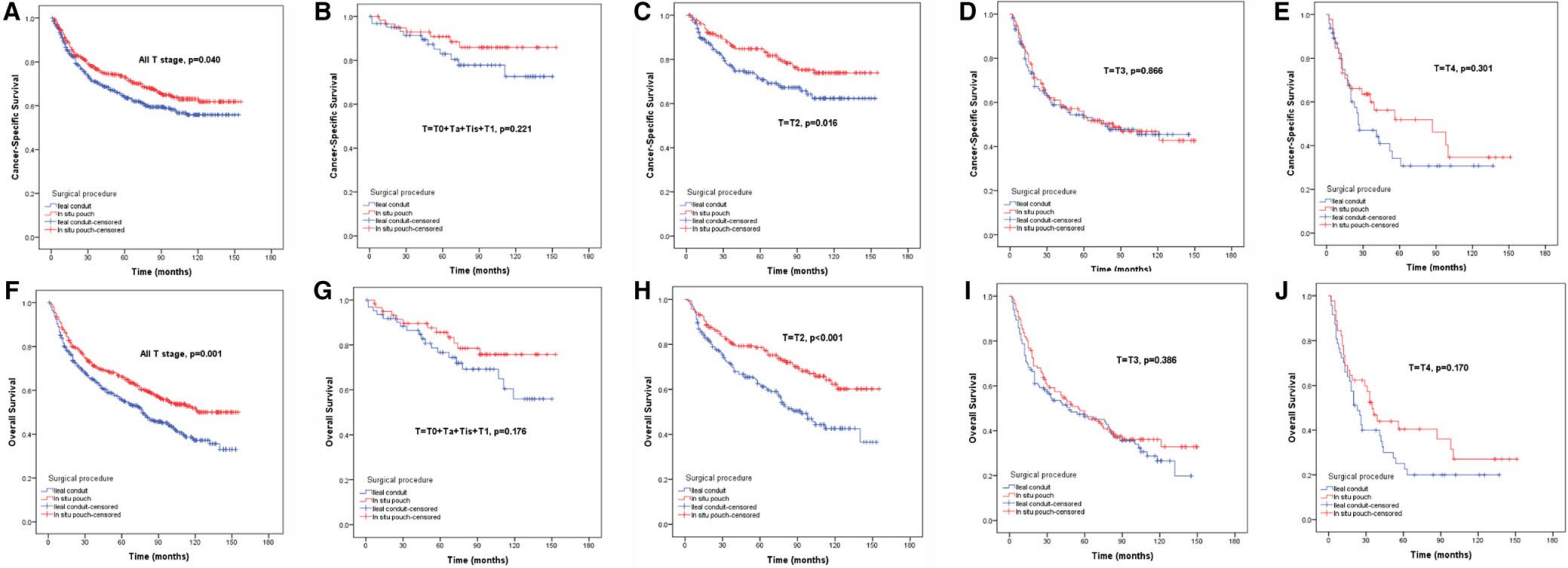
Influence of the urinary diversion on Cancer‐Specific Survival (A‐E) and Overall Survival (F‐J) in Bladder Cancer patients stratified by pathological T stage after propensity score matching

After 1:1 PSM, cox regression analyses showed that ON was a protective factor for CSS and OS for all T stages by multivariate analysis (ON vs ID, HR: 0.741, 95% CI: 0.583‐0.941, *P* = .014; HR: 0.671, 95% CI: 0.548‐0.823, *P* < .001, respectively) (Table 2, Figure 3A and Table 3, Figure 3F respectively). However, when stratified by T stages, subgroup analysis revealed that only patients with pathological T2 stage obtained survival benefit from ON after PSM in terms of CSS (*P* = .016) and OS (*P* < .001) (Figure 3B‐3E and Figure 3G‐3J).

**TABLE 2:** Univariate and multivariate regression analyses for CSM after PSM

Characteristic	Univariate			Multivariate		
	HR	95% CI	*P*	HR	95% CI	*P*
Age	1.015	1.002‐1.028	.019*	1.012	0.999‐0.025	.073*
Gender						
Female	Ref.		.040	Ref.		.077
Male	0.497	0.255‐0.968		0.542	0.274‐1.069	
Race						
White	Ref.		.499			
Black	1.077	0.604‐1.922	.801			
AI	0.750	0.105‐5.344	.774			
API	0.546	0.281‐1.062	.075			
Unknown	0.001	0.001‐1.003	.946			
Marital status						
Married	Ref.		.179			
No	1.229	0.944‐1.601	.125			
Unknown	0.373	0.052‐2.662	.325			
Primary tumor site						
Trigone of bladder	Ref.		.938			
Dome of bladder	0.788	0.372‐1.668	.533			
Lateral wall of bladder	0.833	0.482‐1.439	.512			
Anterior wall of bladder	0.331	0.077‐1.427	.138			
Posterior wall of bladder	0.980	0.532‐1.806	.949			
Bladder neck	0.598	0.222‐1.610	.309			
Ureteric orifice	0.917	0.310‐2.710	.875			
Urachus	0.001	0.001‐1.002	.948			
Overlapping lesion of bladder	0.863	0.508‐1.468	.588			
Bladder, NOS	0.896	0.544‐1.475	.666			
Histology						
Transitional cell carcinoma	Ref.		.005*	Ref.		.077
Squamous cell carcinoma	0.415	0.133‐1.294	.130	0.421	0.134‐1.326	.139
Other types	1.956	1.227‐3.119	.005*	1.583	0.919‐2.727	.098
Pathological T stage						
T0‐Tis‐Ta‐T1	Ref.		<.001*	Ref.		<.001*
T2	1.737	1.060‐2.847	.028*	1.618	0.986‐2.655	.057
T3	4.020	2.469‐6.545	<.001*	3.066	1.857‐5.062	<.001*
T4	5.442	3.199‐9.257	<.001*	4.315	2.510‐7.419	<.001*
Tx/NA	4.203	1.430‐12.358	.009*	2.915	0.370‐22.986	.310
Pathological N stage						
N0	Ref.		<.001*	Ref.		<.001*
N1	2.854	2.083‐3.910	<.001*	1.990	1.432‐2.765	<.001*
N2	3.299	2.351‐4.628	<.001*	2.615	1.843‐3.711	<.001*
N3 (n = 0)						
Nx/NA	2.067	0.850‐5.030	.109	1.454	0.237‐8.905	.686
Pathological M stage						
M0	Ref.		.012*	Ref.		.673
M1	2.257	1.317‐3.868	.003*	1.067	0.599‐1.903	.826
Mx/NA	1.244	0.463‐3.340	.665	0.270	0.014‐5.228	.387
Grade						
I	Ref.		.477			
II	0.650	0.138‐3.059	.585			
III	1.064	0.262‐4.329	.931			
IV	1.190	0.295‐4.797	.807			
Unknown	1.311	0.296‐5.811	.721			
Lymphadenectomy						
No	Ref.		.906			
Yes	1.030	0.486‐2.182	.939			
Others	0.664	0.082‐5.396	.702			
Urinary diversion						
Ileal conduilt	Ref.		.042*	Ref		.014*
Orthotopic neobladder	0.781	0.616‐0.991		0.741	0.583‐0.941	
Adjuvant radiation						
No	Ref.		.003*	Ref.		.070
Yes	3.864	1.589‐9.393		2.389	0.932‐6.126	
Adjuvant chemotherapy						
No	Ref.		.011*	Ref.		.395
Yes	1.362	1.073‐1.728		0.890	0.681‐1.164	

Abbreviations: AI, American Indian/Alaskan Native; API, Asian/Pacific Islander; CI, confidence interval; CSM, cancer‐specific mortality; HR, hazard ratio; NA, not available; NOS, not otherwise specified; PSM, propensity score matching; Ref., reference.

* Statistically significant.

**TABLE 3:** Univariate and multivariate regression analyses for ACM after PSM

Characteristic	Univariate			Multivariate		
	HR	95% CI	*P*	HR	95% CI	*P*
Age	1.025	1.014‐1.036	<.001*	1.021	1.010‐1.033	<.001*
Gender						
Female	Ref.		.041*	Ref.		.054
Male	0.535	0.293‐0.976		0.551	0.300‐1.012	
Race						
White	Ref.		.172			
Black	1.007	0.610‐1.662	.977			
AI	1.127	0.281‐4.528	.866			
API	0.463	0.254‐0.844	.012*			
Unknown (n = 2)						
Marital status						
Married	Ref.		.032*	Ref.		.100
No	1.315	1.055‐1.639	.015*	1.276	1.015‐1.605	.037*
Unknown	0.536	0.133‐2.154	.379	0.733	0.181‐2.958	.662
Primary tumor site						
Trigone of bladder	Ref.		.813			
Dome of bladder	0.804		.470			
Lateral wall of bladder	0.780		.269			
Anterior wall of bladder	0.416		.101			
Posterior wall of bladder	0.741		.257			
Bladder neck	0.540		.145			
Ureteric orifice	0.597		.334			
Urachus (n = 2)						
Overlapping lesion of bladder	0.726	0.470‐1.120	.148			
Bladder, NOS	0.809	0.541‐1.211	.303			
Histology						
Transitional cell carcinoma	Ref.		.040*	Ref.		.084
Squamous cell carcinoma	0.382	0.143‐1.023	.056	0.381	0.141‐1.029	.057
Other types	1.451	0.925‐2.277	.105	1.331	0.796‐2.227	.276
Pathological T stage						
T0‐Tis‐Ta‐T1	Ref.		<.001*	Ref.		<.001*
T2	1.656	1.123‐2.442	.011*	1.552	1.051‐2.292	.027
T3	3.307	2.243‐4.875	<.001*	2.584	1.732‐3.854	<.001*
T4	4.520	2.939‐6.951	<.001*	3.718	2.394‐5.774	<.001*
Tx/NA	2.642	0.932‐7.487	.068	0.647	0.135‐3.098	.586
Pathological N stage						
N0	Ref.		<.001*	Ref.		<.001*
N1	2.307	1.739‐3.062	<.001*	1.577	1.171‐2.123	.003*
N2	2.885	2.144‐3.882	<.001*	2.401	1.768‐3.261	<.001*
N3 (n = 0)						
Nx/NA	2.007	0.947‐4.252	.069	3.284	1.050‐10.270	.041*
Pathological M stage						
M0	Ref.		.024*	Ref.		.982
M1	1.970	1.211‐3.206	.006*	0.975	0.571‐1.664	.926
Mx/NA	1.127	0.466‐2.724	.791	0.849	0.128‐5.644	.866
Grade						
I	Ref.		.511			
II	0.615	0.204‐1.853	.388			
III	0.746	0.275‐2.023	.564			
IV	0.872	0.325‐2.343	.786			
Unknown	0.822	0.275‐2.459	.726			
Lymphadenectomy						
No	Ref.		.961			
Yes	0.956	0.525‐1.743	.884			
Others	0.806	0.179‐3.639	.780			
Urinary diversion						
Ileal conduit	Ref.		.001*	Ref.		<.001*
Orthotopic neobladder	0.705	0.576‐0.862		0.671	0.548‐0.823	
Adjuvant radiation						
No	Ref.		.002*	Ref.		.033*
Yes	3.523	1.567‐7.919		2.526	1.080‐5.908	
Adjuvant chemotherapy						
No	Ref.		.098			
Yes	1.187	0.969‐1.455				

Abbreviations: ACM, all‐cause mortality; AI, American Indian/Alaskan Native; API, Asian/Pacific Islander; CI, confidence interval; HR, hazard ratio; NA, not available; NOS, not otherwise specified; PSM, propensity score matching; Ref., reference.

* Statistically significant.

## 4 DISCUSSION

RC and urinary diversion are two important steps of the operation for BCa patients. There are different choices of urinary diversions after RC, including uretero‐cutaneostomy, IC, continent cutaneous urinary diversion, ureterocolonic diversion, and ON according to the patients’ preference, performance status, life expectancy, and oncological control. Among these urinary diversions, IC and ON are the most commonly utilized ones in the clinical practice.^2^, ^3^ As a continent diversion, ON offers many advantages and represents an excellent option for urinary diversion for appropriate cases. However, large studies based on the United States and European populations have demonstrated that the utilization of orthotopic diversion has decreased over time.^6^, ^7^ The reason for this phenomenon may be due to increased adoption of robotic radical cystectomy and increased utilization of intracorporeal urinary diversion.^3^ Intracorporeal ON is a difficult technique, and thus, is associated with a higher rate of high‐grade complications.^8^, ^9^ Complications due to urinary diversion can prolong the hospital stay, increase hospitalization expenses, and impact all‐other cause survival. Different kinds of complications related to urinary diversion after RC have been reported, but most are due to the use of the bowel for urinary diversion. Complications related to IC diversion include urinary tract infections, pyelonephritis, ureteroileal leakage and stenosis, and stomal problems.^10^, ^11^ In two studies with large populations, complications associated with ON included diurnal and nocturnal incontinence, ureterointestinal stenosis, metabolic disorders, and vitamin B12 deficiency.^12^, ^13^ One previous retrospective study revealed that there was no significant difference in the decline of estimated glomerular filtration rate between IC and ON in patients with preoperative chronic kidney disease.^14^ In this study, we focus on the impact of IC and ON diversion on the oncological outcomes of BCa patients. Our aim is to provide some decision‐making guidance to urologists and patients regarding urinary diversion.

Consistent with a previous study,^5^ this study showed that the ON cohort comprised younger and male patients and with a significantly higher percentage of early T stages compared to the IC cohort. Age alone is not a criterion for choosing continent ON diversion, but age >80 years is often considered to be the threshold.^2^ However, the optimal cut‐off value for age is still disputed. In our opinion, younger patients with BCa would have longer life expectancy, better tolerance for a complicated orthotopic diversion technique, and easier recovery from postoperational complications. A higher male‐to‐female ratio has also been observed among patients undergoing ON substitution in some large series from experience centers.^15^, ^16^ Male BCa patients are more suitable to receive ON substitution after RC due to the following reasons: (a) the incidence rate of BCa is higher in men than in women (male to female ratio 3:1)^1^; (b) the urethra of men is longer than that of women, which would, to some extent, ensure urinary continence after ON substitution. Frozen sections of pelvic lymph nodes and surgical margins have to be performed during the operation if ON diversion is planned. Positive surgical margins, N2 or N3 disease would exclude orthotopic neobladder reconstruction,^2^, ^3^ which may be the potential reason why early T stages are found in the ON group.

There have been many studies on the impact of ON and IC diversions on the HRQoL of BCa patients, but the influence of ON diversion on cancer control has not been adequately studied. One systemic review, which included 29 studies, found no significant differences in QoL after radical cystectomy between continent and incontinent urinary diversion.^17^ However, by maintaining body image and normal urethral voiding in suitable patients, ON could offer a better QoL. In a relatively large study with 214 ON and 269 IC patients, Yossepowitch and his colleagues demonstrated that ON diversion had a more favorable CSS and OS compared to IC diversion (*P* = .04 and *P* = .001, respectively). However, in the subgroup analysis this statistical significance disappeared when further stratified into organ confined and nonorgan confined diseases.^5^ This was the only study on the impact of ON and IC diversions on oncological outcomes that we could find. In contrast with the above study, this study found better 5‐year CSS and 5‐year OS in the ON group for either all T stages together or T stage subgroups. Furthermore, given the discrepancies between the two groups, we performed 1:1 PSM to adjust for age, gender, sex, marital status, TNM stages, lymphadenectomy, radiotherapy, and chemotherapy. After PSM, multiple cox regression analyses showed that ON was a protective factor for CSS and OS for all T stages. However, when stratified for pathological T stages, subgroup analysis revealed that only patients with pathological T2 stage obtained CSS (*P* = .016) and OS (*P* < .001) benefit from ON diversion.

This study has some limitations as well as some strengths. Among limitations, first, the study was of a retrospective nature, which means that the significant survival outcomes could be the effect of strict selection criteria for ON diversion. As a consequence, a randomized controlled trial to compare the long‐term oncological outcomes between ON diversion and IC diversion is important and urgent. Second, the documentation on lymphadenectomy was not very precise about the scope of lymph node dissection, which may have had an impact on survival outcomes.^18^ Third, there was no documentation on BCa recurrence in the SEER database; thus, we could not analyze recurrence‐free survival. Moreover, we did not control for performance status or any prognostic risk score at baseline. Consequently, as patients who have better performance status may be eligible for ON vs IC, this may result in selection bias. Nonetheless, to the best of our knowledge, the strength of this study is that it is the largest one to compare survival outcomes between the two groups based on the SEER cancer registries, which cover approximately 28% of the population of the United States. Most clinical trials have excluded patients who had a history of other prior malignancy, since this may have an impact on survival outcomes.^19^, ^20^ Based on this, and to ensure the reliability of our results, we also excluded patients with previous tumor history. Finally, the PSM method also increased the accuracy of this study.

## 5 CONCLUSIONS

As a continent diversion, ON diversion showed some advantages in terms of survival outcomes compared to IC diversion. Young, married, and male patients with early pathological T stage, especially T2 stage, were more suitable to receive RC plus ON surgery, which could improve their probability of survival.

## CONFLICT OF INTEREST

The authors declare that they have no competing interests.

## AUTHOR CONTRIBUTIONS

Xiaohong Su: Data curation, formal analysis, investigation, methodology, project administration, writing‐original draft. Kaihui Wu, Shuo wang and Wei Su: Data curation, formal analysis, investigation. Bingkun Li and Chuanyin Li: Data curation, formal analysis, investigation. Xiangming Mao: Conceptualization, project administration, supervision, and writing‐review and editing.

## Supporting information

TableS1‐S3

## Data Availability

The raw data underlying this paper is available upon request to from the corresponding author.
