# Supplementary material for: The impact of orthotopic neobladder vs ileal conduit urinary diversion after cystectomy on the survival outcomes in patients with bladder cancer: A propensity score matched analysis
Source: Cancer Med. 2020 Sep 1;9(20):7590–600. doi: 10.1002/cam4.3404 (PMC7571812; doi:10.1002/cam4.3404)
Supplement: Supplementary file 1 — TableS1‐S3 [file CAM4-9-7590-s001.doc]

| Supplement 1 Clinical and pathological characteristics of BCa patients between different urinary diversion before PSM | | | | |
| --- | --- | --- | --- | --- |
| Characteristic | Urinary diversion, % of patients | | Chi-square or Z | p value |
|  | Ileal conduit  (n=5071) | Orthotopic neobladder  (n=409) |  |  |
| Age, Median (IQR) | 69 (61-75) | 63 (55-69) | -10.796 | <0.001^*^ |
| Sex |  |  | 36.317 | <0.001^*^ |
| Male | 86.9 | 97.1 |  |  |
| Female | 13.1 | 2.9 |  |  |
| Race |  |  | 8.957 | 0.062 |
| White | 89.8 | 89.7 |  |  |
| Black | 5.3 | 2.9 |  |  |
| AI | 0.4 | 0.5 |  |  |
| API | 4.3 | 6.6 |  |  |
| Unknown | 0.2 | 0.2 |  |  |
| Marital status |  |  | 15.609 | <0.001^*^ |
| Married | 64.1 | 73.3 |  |  |
| No | 32.5 | 25.2 |  |  |
| Unknown | 3.4 | 1.5 |  |  |
| Primary tumor site |  |  | 16.204 | 0.063 |
| Trigone of bladder | 6.0 | 3.9 |  |  |
| Dome of bladder | 3.7 | 3.7 |  |  |
| Lateral wall of bladder | 16.5 | 17.1 |  |  |
| Anterior wall of bladder | 3.5 | 2.4 |  |  |
| Posterior wall of bladder | 7.4 | 7.8 |  |  |
| Bladder neck | 2.1 | 3.7 |  |  |
| Ureteric orifice | 1.2 | 1.5 |  |  |
| Urachus | 0.1 | 0.5 |  |  |
| Overlapping lesion of bladder | 21.0 | 20.0 |  |  |
| Bladder, NOS | 38.5 | 39.4 |  |  |
| Histology |  |  | 1.349 | 0.509 |
| Transitional cell carcinoma | 90.7 | 92.2 |  |  |
| Squamous cell carcinoma | 3.7 | 2.7 |  |  |
| Other types | 5.6 | 5.1 |  |  |
| Pathological T stage |  |  | 19.757 | 0.001^*^ |
| T0-Tis-Ta-T1 | 10.9 | 14.4 |  |  |
| T2 | 38.4 | 45.2 |  |  |
| T3 | 32.2 | 28.4 |  |  |
| T4 | 17.3 | 11.0 |  |  |
| Tx/NA | 1.2 | 1.0 |  |  |
| Pathological N stage |  |  | 8.357 | 0.079 |
| N0 | 72.9 | 79.0 |  |  |
| N1 | 13.0 | 9.5 |  |  |
| N2 | 11.9 | 10.0 |  |  |
| N3 | 0.4 | 0.0 |  |  |
| Nx/NA | 1.9 | 1.5 |  |  |
| Pathological M stage |  |  | 1.700 | 0.428 |
| M0 | 94.0 | 94.6 |  |  |
| M1 | 4.1 | 4.4 |  |  |
| Mx/NA | 1.9 | 1.0 |  |  |
| Grade |  |  | 2.977 | 0.562 |
| I | 0.9 | 0.7 |  |  |
| II | 4.6 | 4.2 |  |  |
| III | 30.7 | 34.0 |  |  |
| IV | 58.6 | 57.2 |  |  |
| Unknown | 5.2 | 3.9 |  |  |
| Lymphadenectomy |  |  | 16.435 | <0.001^*^ |
| No | 7.7 | 2.7 |  |  |
| Yes | 91.3 | 97.1 |  |  |
| Others | 0.9 | 0.2 |  |  |
| Adjuvant radiation |  |  | 6.346 | 0.012^*^ |
| No | 96.1 | 98.5 |  |  |
| Yes | 3.9 | 1.5 |  |  |
| Adjuvant chemotherapy |  |  | 0.647 | 0.421 |
| No | 61.4 | 59.4 |  |  |
| Yes | 38.6 | 40.6 |  |  |
| **Abbreviations:** BCa, bladder cancer; PSM, propensity score matching; IQR, interquartile range; AI, American Indian/Alaskan Native; API, Asian/Pacific Islander; NOS, not otherwise specified; NA, not available | | | | |
| **^*^** statistically significant. | | | | |

| Supplement 2 Univariate and multivariate regression analyses for CSM before PSM | | | | | | | |
| --- | --- | --- | --- | --- | --- | --- | --- |
| Characteristic | Univariate | | |  | Multivariate | | |
|  | HR | 95% CI | p |  | HR | 95% CI | p |
| Age | 1.013 | 1.009-1.017 | <0.001^*^ |  | 1.015 | 1.011-1.020 | <0.001^*^ |
| Sex |  |  |  |  |  |  |  |
| Female | Ref. |  | 0.184 |  |  |  |  |
| Male | 0.917 | 0.807-1.042 |  |  |  |  |  |
| Race |  |  |  |  |  |  |  |
| White | Ref. |  | 0.268 |  |  |  |  |
| Black | 1.173 | 0.978-1.407 | 0.085 |  |  |  |  |
| AI | 1.019 | 0.530-1.962 | 0.954 |  |  |  |  |
| API | 0.912 | 0.742-1.122 | 0.385 |  |  |  |  |
| Unknown | 0.518 | 0.167-1.606 | 0.254 |  |  |  |  |
| Marital status |  |  |  |  |  |  |  |
| Married | Ref. |  | <0.001^*^ |  | Ref. |  | <0.001^*^ |
| No | 1.242 | 1.138-1.355 | <0.001^*^ |  | 1.211 | 1.107-1.325 | <0.001^*^ |
| Unknown | 1.331 | 1.608-1.659 | 0.011^*^ |  | 1.565 | 1.251-1.958 | <0.001^*^ |
| Primary tumor site |  |  |  |  |  |  |  |
| Trigone of bladder | Ref. |  | 0.002^*^ |  | Ref. |  | 0.174 |
| Dome of bladder | 0.726 | 0.545-0.968 | 0.029^*^ |  | 0.806 | 0.603-1.076 | 0.143 |
| Lateral wall of bladder | 0.818 | 0.675-0.992 | 0.041^*^ |  | 1.037 | 0.854-1.259 | 0.717 |
| Anterior wall of bladder | 1.008 | 0.770-1.320 | 0.952 |  | 0.985 | 0.750-1.292 | 0.911 |
| Posterior wall of bladder | 0.780 | 0.623-0.977 | 0.031^*^ |  | 0.913 | 0.728-1.146 | 0.433 |
| Bladder neck | 0.921 | 0.673-1.260 | 0.605 |  | 1.117 | 0.813-1.536 | 0.493 |
| Ureteric orifice | 0.748 | 0.486-1.152 | 0.188 |  | 0.968 | 0.628-1.491 | 0.881 |
| Urachus | 0.984 | 0.244-3.972 | 0.982 |  | 1.242 | 0.303-5.097 | 0.764 |
| Overlapping lesion of bladder | 1.049 | 0.874-1.258 | 0.608 |  | 1.077 | 0.896-1.295 | 0.429 |
| Bladder, NOS | 0.970 | 0.815-1.153 | 0.727 |  | 1.123 | 0.942-1.338 | 0.196 |
| Histology |  |  |  |  |  |  |  |
| Transitional cell carcinoma | Ref. |  | <0.001^*^ |  | Ref. |  | 0.006^*^ |
| Squamous cell carcinoma | 1.256 | 1.020-1.546 | 0.032^*^ |  | 1.305 | 1.054-1.616 | 0.015^*^ |
| Other types | 1.437 | 1.223-1.688 | <0.001^*^ |  | 1.231 | 1.034-1.466 | 0.020^*^ |
| Pathological T stage |  |  |  |  |  |  |  |
| T0-Tis-Ta-T1 | Ref. |  | <0.001^*^ |  | Ref. |  | <0.001^*^ |
| T2 | 1.406 | 1.170-1.698 | <0.001^*^ |  | 1.318 | 1.095-1.587 | 0.004^*^ |
| T3 | 3.330 | 2.787-3.980 | <0.001^*^ |  | 2.540 | 2.113-3.053 | <0.001^*^ |
| T4 | 5.108 | 4.247-6.143 | <0.001^*^ |  | 3.370 | 2.775-4.092 | <0.001^*^ |
| Tx/NA | 3.264 | 2.253-4.728 | <0.001^*^ |  | 1.797 | 1.028-3.142 | 0.040^*^ |
| Pathological N stage |  |  |  |  |  |  |  |
| N0 | Ref. |  | <0.001^*^ |  | Ref. |  | <0.001^*^ |
| N1 | 2.512 | 2.254-2.799 | <0.001^*^ |  | 1.959 | 1.744-2.200 | <0.001^*^ |
| N2 | 3.502 | 3.145-3.900 | <0.001^*^ |  | 2.605 | 2.314-2.933 | <0.001^*^ |
| N3 | 7.146 | 4.424-11.544 | <0.001^*^ |  | 3.579 | 2.184-5.862 | <0.001^*^ |
| Nx/NA | 1.897 | 1.441-2.496 | <0.001^*^ |  | 1.113 | 0.714-1.735 | 0.638 |
| Pathological M stage |  |  |  |  |  |  |  |
| M0 | Ref. |  | <0.001^*^ |  | Ref. |  | <0.001^*^ |
| M1 | 3.778 | 3.235-4.412 | <0.001^*^ |  | 2.119 | 1.798-2.496 | <0.001^*^ |
| Mx/NA | 1.472 | 1.109-1.954 | 0.007^*^ |  | 1.509 | 0.986-2.311 | 0.058 |
| Grade |  |  |  |  |  |  |  |
| I | Ref. |  | 0.025^*^ |  | Ref. |  | 0.303 |
| II | 0.808 | 0.497-1.312 | 0.389 |  | 0.828 | 0.508-1.348 | 0.447 |
| III | 1.103 | 0.708-1.720 | 0.664 |  | 0.981 | 0.620-1.551 | 0.933 |
| IV | 1.025 | 0.659-1.594 | 0.913 |  | 0.938 | 0.593-1.483 | 0.785 |
| Unknown | 0.890 | 0.551-1.436 | 0.632 |  | 0.810 | 0.494-1.330 | 0.405 |
| Lymphadenectomy |  |  |  |  |  |  |  |
| No | Ref. |  | <0.001^*^ |  | Ref. |  | <0.001^*^ |
| Yes | 0.684 | 0.594-0.788 | <0.001^*^ |  | 0.540 | 0.465-0.627 | <0.001^*^ |
| Others | 0.840 | 0.531-1.328 | 0.455 |  | 0.583 | 0.366-0.928 | 0.023^*^ |
| Urinary diversion |  |  |  |  |  |  |  |
| Ileal conduit | Ref. |  | <0.001^*^ |  | Ref. |  | <0.001^*^ |
| Orthotopic neobladder | 0.584 | 0.488-0.698 |  |  | 0.692 | 0.576-0.831 |  |
| Adjuvant radiation |  |  |  |  |  |  |  |
| No | Ref. |  | <0.001^*^ |  | Ref. |  | 0.004^*^ |
| Yes | 2.115 | 1.780-2.512 |  |  | 1.303 | 1.087-1.561 |  |
| Adjuvant chemotherapy |  |  |  |  |  |  |  |
| No | Ref. |  | 0.088 |  |  |  |  |
| Yes | 1.075 | 0.989-1.169 |  |  |  |  |  |
| **Abbreviations:** CSM, cancer-specific mortality; PSM, propensity score matching; HR, hazard ratio; CI, confidence interval; Ref., reference; AI, American Indian/Alaskan Native; API, Asian/Pacific Islander; NOS, not otherwise specified; NA, not available | | | | | | | |
| ^*^ statistically significant. | | | | | | | |

| Supplement 3 Univariate and multivariate regression analyses for ACM before PSM | | | | | | | |
| --- | --- | --- | --- | --- | --- | --- | --- |
| Characteristic | Univariate | | |  | Multivariate | | |
|  | HR | 95% CI | p |  | HR | 95% CI | p |
| Age | 1.024 | 1.020-1.028 | <0.001**^*^** |  | 1.025 | 1.022-1.029 | <0.001**^*^** |
| Sex |  |  |  |  |  |  |  |
| Female | Ref. |  | 0.479 |  |  |  |  |
| Male | 0.960 | 0.858-1.075 |  |  |  |  |  |
| Race |  |  |  |  |  |  |  |
| White | Ref. |  | 0.018**^*^** |  | Ref. |  | 0.007**^*^** |
| Black | 1.210 | 1.038-1.411 | 0.015**^*^** |  | 1.124 | 0.961-1.314 | 0.143 |
| AI | 1.045 | 0.606-1.802 | 0.874 |  | 1.000 | 0.578-1.730 | 1.000 |
| API | 0.867 | 0.724-1.037 | 0.119 |  | 0.813 | 0.679-0.974 | 0.025**^*^** |
| Unknown | 0.365 | 0.118-1.133 | 0.081 |  | 0.229 | 0.074-0.713 | 0.011**^*^** |
| Marital status |  |  |  |  |  |  |  |
| Married | Ref. |  | <0.001**^*^** |  | Ref. |  | <0.001**^*^** |
| No | 1.288 | 1.196-1.386 | <0.001**^*^** |  | 1.280 | 1.188-1.380 | <0.001**^*^** |
| Unknown | 1.322 | 1.093-1.598 | 0.004**^*^** |  | 1.606 | 1.328-1.944 | <0.001**^*^** |
| Primary tumor site |  |  |  |  |  |  |  |
| Trigone of bladder | Ref. |  | 0.011**^*^** |  | Ref. |  | 0.143 |
| Dome of bladder | 0.807 | 0.635-1.025 | 0.079 |  | 0.857 | 0.673-1.092 | 0.212 |
| Lateral wall of bladder | 0.890 | 0.755-1.050 | 0.166 |  | 1.069 | 0.905-1.262 | 0.435 |
| Anterior wall of bladder | 1.091 | 0.868-1.372 | 0.453 |  | 1.056 | 0.838-1.330 | 0.647 |
| Posterior wall of bladder | 0.842 | 0.695-1.020 | 0.079 |  | 0.956 | 0.788-1.159 | 0.645 |
| Bladder neck | 0.896 | 0.681-1.181 | 0.436 |  | 1.085 | 0.821-1.434 | 0.566 |
| Ureteric orifice | 0.859 | 0.605-1.220 | 0.396 |  | 1.082 | 0.760-1.539 | 0.662 |
| Urachus | 0.745 | 0.185-3.001 | 0.679 |  | 1.123 | 0.275-4.585 | 0.871 |
| Overlapping lesion of bladder | 1.058 | 0.903-1.238 | 0.486 |  | 1.085 | 0.925-1.273 | 0.315 |
| Bladder, NOS | 1.009 | 0.868-1.172 | 0.910 |  | 1.141 | 0.981-1.329 | 0.088 |
| Histology |  |  |  |  |  |  |  |
| Transitional cell carcinoma | Ref. |  | <0.001**^*^** |  | Ref. |  | 0.003**^*^** |
| Squamous cell carcinoma | 1.233 | 1.034-1.471 | 0.020**^*^** |  | 1.251 | 1.047-1.495 | 0.014**^*^** |
| Other types | 1.322 | 1.146-1.524 | <0.001**^*^** |  | 1.213 | 1.043-1.410 | 0.012**^*^** |
| Pathological T stage |  |  |  |  |  |  |  |
| T0-Tis-Ta-T1 | Ref. |  | <0.001**^*^** |  | Ref. |  | <0.001**^*^** |
| T2 | 1.284 | 1.116-1.478 | <0.001**^*^** |  | 1.204 | 1.046-1.387 | 0.010**^*^** |
| T3 | 2.652 | 2.310-3.045 | <0.001**^*^** |  | 2.091 | 1.815-2.409 | <0.001**^*^** |
| T4 | 3.772 | 3.261-4.363 | <0.001**^*^** |  | 2.650 | 2.276-3.087 | <0.001**^*^** |
| Tx/NA | 2.384 | 1.724-3.297 | <0.001**^*^** |  | 1.437 | 0.894-2.308 | 0.134 |
| Pathological N stage |  |  |  |  |  |  |  |
| N0 | Ref. |  | <0.001**^*^** |  | Ref. |  | <0.001**^*^** |
| N1 | 2.046 | 1.858-2.252 | <0.001**^*^** |  | 1.672 | 1.512-1.851 | <0.001**^*^** |
| N2 | 2.866 | 2.602-3.157 | <0.001**^*^** |  | 2.277 | 2.052-2.527 | <0.001**^*^** |
| N3 | 5.434 | 3.413-8.653 | <0.001**^*^** |  | 3.073 | 1.918-4.925 | <0.001**^*^** |
| Nx/NA | 1.622 | 1.274-2.065 | <0.001**^*^** |  | 1.074 | 0.734-1.573 | 0.713 |
| Pathological M stage |  |  |  |  |  |  |  |
| M0 | Ref. |  | <0.001**^*^** |  | Ref. |  | <0.001**^*^** |
| M1 | 3.176 | 2.747-3.672 | <0.001**^*^** |  | 1.956 | 1.682-2.275 | <0.001**^*^** |
| Mx/NA | 1.374 | 1.072-1.760 | 0.012**^*^** |  | 1.501 | 1.043-2.160 | 0.029**^*^** |
| Grade |  |  |  |  |  |  |  |
| I | Ref. |  | 0.034**^*^** |  | Ref. |  | 0.411 |
| II | 0.880 | 0.586-1.321 | 0.536 |  | 0.921 | 0.612-1.386 | 0.692 |
| III | 1.099 | 0.755-1.600 | 0.621 |  | 1.027 | 0.699-1.511 | 0.890 |
| IV | 1.034 | 0.712-1.501 | 0.863 |  | 0.988 | 0.672-1.452 | 0.951 |
| Unknown | 0.896 | 0.598-1.344 | 0.596 |  | 0.878 | 0.579-1.332 | 0.541 |
| Lymphadenectomy |  |  |  |  |  |  |  |
| No | Ref. |  | <0.001**^*^** |  | Ref. |  | <0.001**^*^** |
| Yes | 0.694 | 0.614-0.783 | <0.001**^*^** |  | 0.590 | 0.521-0.669 | <0.001**^*^** |
| Others | 0.876 | 0.598-1.284 | 0.498 |  | 0.618 | 0.421-0.908 | 0.014**^*^** |
| Urinary diversion |  |  |  |  |  |  |  |
| Ileal conduit | Ref. |  | <0.001**^*^** |  | Ref. |  | <0.001**^*^** |
| Orthotopic neobladder | 0.542 | 0.465-.633 |  |  | 0.677 | 0.579-.793 |  |
| Adjuvant radiation |  |  |  |  |  |  |  |
| No | Ref. |  | <0.001**^*^** |  | Ref. |  | 0.006**^*^** |
| Yes | 1.868 | 1.596-2.185 |  |  | 1.255 | 1.067-1.476 |  |
| Adjuvant chemotherapy |  |  |  |  |  |  |  |
| No | Ref. |  | 0.080 |  |  |  |  |
| Yes | 0.938 | 0.872-1.008 |  |  |  |  |  |
| **Abbreviations:** ACM, all-cause mortality; PSM, propensity score matching; HR, hazard ratio; CI, confidence interval; Ref., reference; AI, American Indian/Alaskan Native; API, Asian/Pacific Islander; NOS, not otherwise specified; NA, not available | | | | | | | |
| **^*^** statistically significant. | | | | | | | |
